# Fertility preservation in BRCA mutation carriers—efficacy and safety issues: a review

**DOI:** 10.1186/s12958-019-0561-0

**Published:** 2020-02-18

**Authors:** Xiaofu Zhang, Jingxin Niu, Tuanjie Che, Yibei Zhu, Hongtao Zhang, Jing Qu

**Affiliations:** 1 Department of Clinical Medicine, Medical College of Soochow University, Ren Ai Road 199, Suzhou Industrial Park, Suzhou, 215123 China; 2 Laboratory of Precision Medicine and Translational Medicine, Suzhou Hospital Affiliated to Nanjing Medical University, Suzhou Science and Technology Town Hospital, Suzhou, 215153 China; 3 Department of Immunology, Medical College of Soochow University, Ren Ai Road 199, Suzhou Industrial Park, Suzhou, 215123 China; 4 Department of Orthopedics, the First Affiliated Hospital of Soochow University, Soochow University, Suzhou, 215006 China; 5 Department of Cell Biology, Medical College of Soochow University, Ren Ai Road 199, Suzhou Industrial Park, Suzhou, 215123 China

**Keywords:** Fertility preservation, BRCA mutation, Infertility, In vitro fertilization

## Abstract

BRCA mutation carriers face various situations that influence their fertility potential. There is still a lack of guideline or expert consensus on Fertility Preservation (FP) in BRCA mutation carriers and the necessity and safety of FP in BRCA mutation carriers is still in dispute. This review aims to focus on the population of BRCA mutation carriers by analyzing the existing FP strategies, comprehensively comparing the pros and cons of each strategy and its applicability.

FP is a suggestion for BRCA mutation carriers with birth planning. Different FP strategies have different characteristics. Considering the particularity of BRCA mutation carriers, multiple factors need to be carefully considered. This review focuses on the applicability of each FP method for carriers under various circumstances. Available FP strategies including oocyte cryopreservation, ovarian tissue cryopreservation, preimplantation genetic diagnosis, and egg/embryo donation are analyzed by comparing existing methods comprehensively. In the attempt to provide an up-to-date decision-making guidance. Conditions taking into consideration were the carrier’s age, the risk of breast and ovarian metastasis, plans for oncotherapy, FP outcome, time available for FP intervention and accessibility.

Overall, FP is necessary and safe for BRCA mutation carriers. Among all available FP methods, oocyte cryopreservation is the most reliable procedure; ovarian tissue cryopreservation is the only way for preserving both fertility and endocrine function, recommended for pre-pubertal carriers and when time is limited for oocyte stimulation. A clear framework provides frontline clinical practitioners a new thought and eventually benefit thousands of BRCA mutation carriers.

## Background

The BRCA gene is an important tumor suppressor gene. It is generally believed that BRCA gene mutation is an important factor leading to Hereditary Breast and Ovarian Cancer Syndrome (HBCD). BRCA mutation carriers have a lifetime risk of breast cancer of 69–72%, and carriers are 10 to 30 times more likely to develop ovarian cancer than non-carriers. Meanwhile, BRCA mutation carriers face many conditions that may affect their fertility. On the one hand, studies have shown that BRCA mutations are associated with Premature Ovarian Failure (POF); on the other hand, some treatments related to BRCA mutations, such as estrogen replacement therapy and prophylactic bilateral salpingo-oophorectomy, also have adverse effects on their fertility potential. Therefore, Fertility Preservation (FP) is of clinical significance for BRCA mutation carriers with birth planning. Currently, FP strategies available include oocyte cryopreservation, Ovarian Tissue Cryopreservation (OTC), Preimplantation Genetic Diagnosis (PGD) before embryo transfer, and egg/embryo donation. Oocyte cryopreservation after Controlled Ovarian Stimulation (COS) is now the most reliable method for FP in post-pubertal women, but COS requires not only a long cycle but also the use of Follicle Stimulating Hormone (FSH) and other hormones that may interfere with the tumor treatment plan of BRCA mutation carriers, and even induce breast and ovarian cancer. Different FP strategies have different characteristics. Considering the particularity of BRCA mutation carriers, factors such as age, breast or ovarian cancer risk and tumor treatment plan need to be carefully considered when selecting FP strategies. Although breast cancer patients have been considered as an adaptive population for FP in some guidelines, there is still a lack of guidelines or expert consensus on FP in carriers of BRCA mutations. There is not enough relevant study taking into account the special circumstances of BRCA mutation carriers by analyzing the feasibility and precautions of FP for them. This review aims to focus on the population of BRCA mutation carriers by analyzing the existing FP strategies, comprehensively comparing the pros and cons of each strategy and its applicability.

## Main text

### Introduction

BRCA1 and BRCA2 are tumor-suppressor genes located on chromosomes 17q21 and 13q12, respectively [1, 2]. Thousands of mutations in either of BRCA1 gene or BRCA2 gene have been identified. Both BRCA1 gene and BRCA2 gene are tumor suppressor genes involving in DNA double-strand break repair and DNA damage-induced checkpoint activation [3]. Tumorigenesis in germline BRCA1/2 pathogenic mutation carriers generally follows a two-hit hypothesis, the first ‘hit’ owing to the inherited pathogenic mutation of one BRCA allele and the second ‘hit’ owing to the somatic inactivation of the second-wild-type allele [4–6]. Alterations of the BRCA1 and BRCA2 genes may also occur through mechanisms other than germline mutations, for example, somatic mutations or epigenetic silencing in sporadic (non-hereditary) EOCs [7]. Among various mutation patterns, some have been determined to be harmful, while others have no proven influence. Online mutation databases, the Breast Cancer Information Core and the BRCA Share™ for example, have identified and classified pathogenic mutations. Chance of inheriting the mutated gene from the mutation carrier parent is 50% for each child [8]. One of the harmful effects BRCA mutation has on carriers is producing hereditary breast cancer and ovarian cancer [9, 10]. While hereditary genetic mutations lead to approximately 10 to15% of breast cancer cases [11, 12], the mutations in BRCA1 and BRCA2 (BRCA) genes are the most penetrating mutations that cause breast cancer [2, 13]. Although only 5 to10% of breast cancer cases in women can be ascribed to BRCA1 or BRCA2 mutations (with BRCA1 mutations slightly more common than BRCA2 mutations), impacts of the gene mutation on carriers is more profound. Pathogenic BRCA1 mutation carriers have a 72% lifetime risk of developing breast cancer, while the risk for BRCA2 mutation carriers is 69% [11]. Women with harmful BRCA mutation have a risk of breast cancer about five times more than non-carriers, and a risk of ovarian cancer about ten to thirty times normal [14]. The risk of breast and ovarian cancer is higher for high-risk BRCA1 mutation carriers than high-risk BRCA2 mutation carriers [15, 16]. Moreover, BRCA mutation accounts for 17 to 65.5% of breast cancer [17, 18] and 16.2 to 40% of ovarian cancer [19, 20]. In addition, BRCA mutation can also increase the possibility of other cancer occurrence, for example, colon cancer, pancreatic cancer, and prostate cancer.

BRCA mutation affect female carriers’ fertility potential directly as it is related to premature ovarian failure (POF). It has been hypothesized that BRCA mutation carriers, especially BRCA1 mutation carriers, are correlated with decreased ovarian reserve, increased fertility-related problems and primary ovarian insufficiency. These can all lead to infertility and early menopause [21–24]. Cumulative evidence has shown that BRCA mutation negatively affect carriers’ ovarian reserve and accelerate ovarian aging, impacting reproductive outcomes both quantitatively and qualitatively. Laboratory and clinical evidence show that BRCA mutation negatively affect carriers’ ovarian reserve. Based on convincing evidence from in vivo results and prospective studies, women with BRCA1 mutation show accelerated ovarian aging due to function of the intact gene decline. This occurs at an earlier age compared to those with BRCA2 mutation [21, 25]. While BRCA1 and BRCA2 are crucial members of the ataxia-telengiectasia mutated (ATM) -mediated double strand break (DSB) repair family of genes, impaired ATM mediated DSB repair act as a cause of aging in human oocytes [26]. When it comes to fertility preservation (FP), some studies have shown that BRCA mutation carriers have a lower number of mature oocytes after ovarian stimulation and a lower follicle reservation. The mean oocyte yield number of BRCA mutation carriers is also lower than non-carriers [25]. Moreover, studies show BRCA mutation carriers have higher rates of low ovarian response compared with BRCA mutation–negative patients undergoing ovarian hyperstimulation [25, 27]. Several studies have demonstrated that asymptomatic BRCA mutation carriers [28–30] as well as breast cancer patients with BRCA mutation [31] have a significantly decreased serum anti-Müllerian hormone (AMH) level, a biomarker representing a woman’s reproductive competence [32]. Low AMH serum concentrations have not been shown to affect natural fecundability and fertility in BRCA mutation carriers below 30 years old, but it does affect those older [33].

**Fig. 1:**
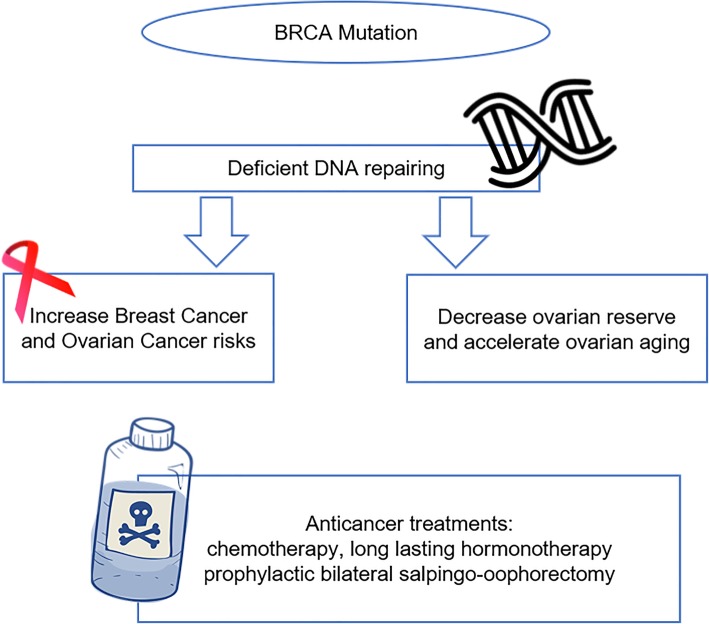
BRCA mutation and it’s negative impact on reproductive potential

Besides direct influences of BRCA mutation on carriers’ fertility capacity, some procedures bounded up with mutation carriers do influence fertility indirectly. Women carriers have a specifically increased lifetime risk of developing breast and tubo-ovarian cancer. Moreover, in order to reduce cancer risk or treat existing malignancy, BRCA mutation carriers are at higher risk of undergoing POF due to medical interventions performed. Tamoxifen, an estrogen antagonist for the primary prevention of breast cancer, is related to treatment-induced POF [34]. For asymptomatic BRCA mutation carriers, their choices of pregnancy and other fertility issues are often influenced by the need of prophylactic bilateral salpingo-oophorectomy at young age [35]. For BRCA mutation carriers who have breast cancer at a young age, antitumor treatments including chemotherapy as well as long-lasting hormonotherapy are significantly associated with ovarian toxicity. These antitumor treatments, given chemotherapy or tamoxifen, increase the apoptosis of follicular reserve [33]. What’s more, the treatments require BRCA mutation carriers delaying pregnancy for several years and some have irreversible harms on fertility [36]. Thus, the direct and indirect impacts of BRCA mutation on female carrier’s fertility potential and capacity are of great significance (Fig. 1). It is important to keep aware of the impacts because this is of clinical importance for BRCA mutation carriers who have childbearing plans. Thus, the recommendation of FP is of necessity and can be applied clinically.

This paper demonstrates the necessity and analyses the safety issues of FP for BRCA mutation carriers. By comparing available FP methods including oocyte cryopreservation, ovarian tissue cryopreservation (OTC), and egg or embryo donation comprehensively, this review provides an up-to-date decision-making guidance. Preimplantation genetic diagnosis (PGD) is introduced as a solution to screen BRCA Gene mutation in embryos. The recommendatory timeline and analysis of adverse effects of FP is also discussed. Undergoing PGD, the choice of hormone for ovarian stimulation and psychosocial evaluation are special considerations suggested during FP for woman with BRCA mutation.

### BRCA mutation screening

#### BRCA germline mutations in different ethnic population

Germline mutations in BRCA1 and BRCA2 genes have important implications in developing appropriate risk assessment and fertility preservation strategies for BRCA mutation carriers. Data support that different BRCA mutation germline confer different risks in breast and ovarian cancer [13]. Since BRCA frequencies vary between populations, understanding population specific BRCA gene distributions can be helpful in identifying mutation carriers [37]. Recent studies have suggested that the variation in human BRCA could be ethnic-specific in different ethnic populations. We used Portuguese population and Chinese population as examples in the following context.

According to research, the c.156_157insAlu BRCA2 rearrangement is a Portuguese founder mutation which is originated about 558 ± 215 years ago. This mutation accounts for the majority of the BRCA2 mutations. Moreover, about one-third of all detrimental germline mutations in Portuguese hereditary breast/ovarian cancer (HBOC) families are related to this mutation. The risk of breast cancer in c.156_157insAlu BRCA2 mutation carriers does not differ from that of other BRCA2 and BRCA1 pathogenic mutation carriers, this is supported by cumulative evidences [38]. Despite the main mutation c.156_157insAlu BRCA2 rearrangement, 2088C > T, 2156delinsCC, and 4255_4256delCT in BRCA1 and 4608_4609delTT, 5036delA, 5583_5584insT, and 8923C > T in BRCA2 are seven new pathogenic point mutations in Portuguese. The new 2156delinsCC was discovered in three probands from different families. Researchers consider it might represent a founder mutation in Portuguese population [39]. In addition to the seven pathogenic mutations, 19 missense mutations of uncertain pathogenic significance were also identified [39].

In Chinese population, c.5154G > A and c.5468-1del8 were two recurrent BRCA1 mutations identified as putative founder mutations [40, 41]. A total of 23 deleterious mutations were detected in the BRCA genes in a study analyzing 133 unrelated Chinese women with familial breast/ovarian cancer living in Zhejiang, eastern China. Five novel deleterious mutations (c.3295delC, c.3780_3781delAG, c.4063_4066delAATC, c.5161 > T and c.5173insA) in BRCA1 and seven (c.1-40delGA, c.4487delC, c.469_473delAAGTC, c.5495delC, c.6141 T > A, c.6359C > G and c.7588C > T) in BRCA2, were identified. The study also found six recurrent mutations and 11 unclassified variants [42, 43]. According to another study based on the population in Shanghai, frameshift mutation was the predominant type in all BRCA mutations, followed by splice site mutation and nonsense mutation [44]. BRCA1/2 mutation germline type and rate vary widely in different populations in China. The current understanding of BRCA mutation pattern in China can only explain a small part of Chinese population [45].

#### BRCA mutation screening methods

BRCA mutation screening by various analytical methods and technology platforms is widely provided by numerous clinical diagnostic laboratories all over the world. Several laboratory practices have been applied [46], such as the arise of treatment-focused genetic testing [47] and the rapid prevalence of next-generation sequencing (NGS) technologies [48, 49]. It is of significance that the result of the BRCA mutation test is accurate, as important clinical decisions are being made based on the results. A comparative study of germline BRCA mutation screening methods in use in European clinical diagnostic laboratories compared the accuracy of NGS, Sanger sequencing, denaturing high-performance liquid chromatography (dHPLC) and high-resolution melting (HRM), the concordance was high (> 97%) across all laboratories using different techniques [50]. Besides genetic testing methods, The Breast Cancer Genetics Referral Screening Tool (B-RST™) was created and validated to identify individuals at increased risk for hereditary breast and ovarian cancer for referral to cancer genetics services easily [51]. It consists of a simple questions used to record both patient responses to family history questions and personal cancer history [52]. It Recent study shows B-RST™ version 3.0 exhibit high sensitivity for BRCA1/2 mutations which remains a simple and quick screening tool for at-risk individuals [53, 54].

### Optional strategies for fertility preservation of BRCA mutation carriers

#### Oocyte cryopreservation

Oocyte cryopreservation after controlled ovarian hyperstimulation (COH) or controlled ovarian stimulation (COS) represents the most established and reliable method for female FP after pubertal onset. From a safety standpoint, fertility treatments have not been associated with an increased risk of gynecologic or breast cancers, even in asymptomatic BRCA mutation carriers [55]. COH is a procedure in the IVF process to obtain more mature eggs since even in the best of circumstances, not every egg typically fertilizes and results in an embryo that develops appropriately. COH uses a variety of drugs and hormones which contain follicle stimulating hormone (FSH) to stimulate follicles grow and allowing doctors to collect more than one mature egg. COH has developed diversified individualized protocols taking age, past history, ovarian reserve testing results (FSH, estradiol, AMH levels), and antral follicle count into account [56]. However, some studies have shown that BRCA mutation carriers have a lower number of mature oocytes after ovarian stimulation and have a lower follicle reservation. BRCA mutation carriers also have a lower mean oocyte yield compared to non-carriers [21, 25]. This conclusion is very controversial, and despite the fact that carriers have a reduced performance, a reasonable ovarian response can still be expected. Oocyte cryopreservation forms an important fertility preservation strategy for BRCA mutation carriers [36].

Despite the advantages of oocyte cryopreservation as a well-established procedure, oocyte cryopreservation has its limitations (Table 1). To begin with, pre-pubertal carriers are not suitable for oocyte cryopreservation since oocyte cryopreservation requires COH which should be performed on reproductively mature carriers [57]. Moreover, COH can be time consuming as it requires 2–5 weeks for COS [58]. Recent studies have shown stimulating the patient regardless of her menstrual-cycle phase, which is defined as random-start COS, has outcomes similar to conventional early follicular phase-start COS for fertility preservation in cancer patients [59, 60]. This protocol would minimize COS cycle to 2–3 weeks, but still time-consuming. Additionally, the stability of oocyte chromosome during oocyte cryopreservation can be affected by the low temperature during cryopreservation regarding to potential impact on chromosome remodeling [61, 62]. This is related to oocyte’s susceptibility to disruption during meiosis, especially during meiotic spindle configuration [63].

**Table 1:** Comparison of oocyte cryopreservation and ovarian tissue cryopreservation

	Advantages	Disadvantages	Recommended applications
Oocyte Cryopreservation	1) Well-established 2) Cost-efficiently and easily-approached 3) No need for surgery	1) Only for reproductively mature carriers 2) Time-consuming 3) Cannot restore endocrine function	Reproductively mature carriers with abundant time
Ovarian Tissue Cryopreservation	1) Restoration of endocrine function 2) Available for pre-puberty carriers 3) Menstrual cycle independent and no need for delay in oncologic treatment	1) Experimental and available only in highly specialized centers 2) Success highly dependent on ovarian reserve 3) Risk of ovarian cancer	Pre-puberty carriers; carriers with adjunct therapy planned or already in therapy

#### Ovarian tissue cryopreservation (OTC)

For woman planning adjuvant chemotherapy or other treatment that might comprise their ovary function, ovarian tissue cryopreservation (OTC) can be a choice to perverse both the fertility function and the endocrine function of the ovary [64, 65]. According to a research based on 20 cases, the success rate of restoration of hormone activity in the ovary is 94%. Moreover, OTC is the only fertility preservation strategy currently available that can also preserve ovarian endocrine function [66].

**Fig. 2:**
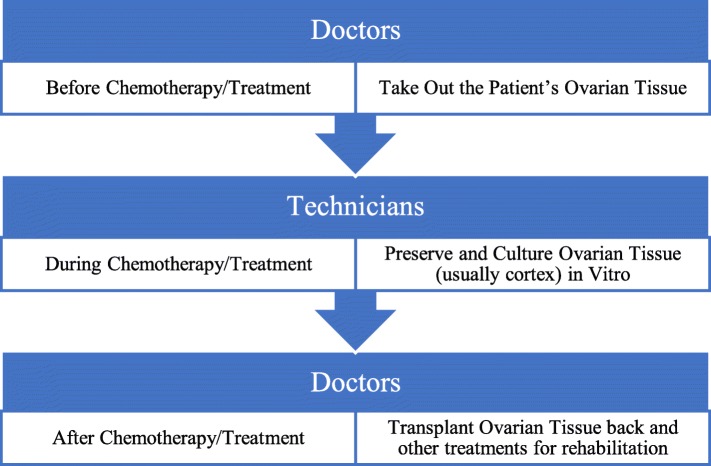
Flow chart of OTC procedure

OTC is done in the following procedure: In order to preserve the patient’s ovarian function, doctors take out part of the ovarian tissue and technicians preserve it in vitro (Fig. 2). Technicians cut the ovarian tissue into slices and culture them in vitro. Technicians usually keep the cortex of the ovary where most of the primary follicles exist. After the therapy, doctors transport the ovarian tissue back to the patient, either to the other side of ovary left or somewhere else, which includes muscle, fallopian tube, the remaining of the ovary that is removed away and so on. This is consistent with current acknowledged procedures [55].

Oocyte cryopreservation is still the most mature choice for FP despite the fact that OTC has been done in many places in Europe and hundreds of babies has born in this way. OTC is only suggested when the therapy is urgent or the condition is not suitable for ovarian stimulation, for example carriers with breast cancer.

At present, embryo and oocyte cryopreservation has been accepted and applied clinically worldwide. However, OTC has not been endorsed by the American Society of Reproductive Medicine and is still considered experimental. Initially, reports of successful human ovarian transplantation cases are few and the success rate remains low. In 2015, OTC has brought 60 babies live and has a worldwide live birth rate of over 30 to 70% [66]. A recent study reported evaluating the long-term follow-up of ovarian tissue cryopreservation followed by auto-transplantation with a live birth rate of 57%, supporting OTC as an effective method to restore fertility [67]. According to a 20-year multicenter investigation, among 46 women undergone OTC, 17 children have been given to birth and all of them are healthy [68]. In the opinion of many pioneers, there is now enough evidence to support OTC and to stop considering it experimental or investigational [66, 69].

There are two methods for OTC: Slow Freezing and Vitrification. Slow Freezing has been the conventional technique for years and Vitrification is commonly used for cryopreservation of embryos and oocytes. Slow Freezing can lead to extensive loss of the follicular pool and excessive damage to stromal cells according to reports. But only two live births have been reported after Vitrification of ovarian tissue by 2015 [70]. Based on data available, present analysis suggests that Vitrification may be more effective than Slow Freezing for OTC, resulting in with fewer primordial follicular DNA strand breaks and better preservation of stromal cells [71]. This should improve ovarian tissue function after transplantation. Vitrification in cryopreservation of ovarian tissue is now an increasingly interest focus area for investigation [72].

According to a 12-years retrospective analysis that evaluated surgical risks, OTC can be considered as an efficient option to preserve the fertility function of children and young adults facing gonadotoxic therapies. Though OTC may introduce the risk of transplanting cancer cells from the transplanted ovarian tissues [73]. Preliminary experience of OTC did not reveal increased risk of cancer relapse. Only three relapses occurred in a study of 32 woman undergone OTC and the relapses were unlikely to be due to OTC [74]. Based on the literature reviews, several procedures including preoperative imaging, histological studies and polymerase chain reaction (PCR) of ovarian tissue can be done before transplantation to identify malignant cells [75]. However, alternative procedures such as oocyte or embryo cryopreservation should still be considered as first options [69]. Because the success of OTC is highly dependent on the patient’s ovarian reserve, several factors such as age and ovarian pathological changes that might affect ovarian reserve influences OTC outcomes. For older carriers or carriers with high risk of neoplastic cells within their ovaries, OTC may not be a proposed choice. Therefore, OTC is suggested for carriers younger than 35 years who normally have a high number of primordial follicles.

As a FP method, OTC has three advantages. Firstly, OTC is now the only FP procedure that can preserve the endocrine function of the ovary as well as its reproductive function. The mean duration of ovarian endocrine function after transplantation is 5 years [76] and the endocrine restoration rate was 63.9% [77]. The restoration of endocrine function can improve the carrier’s pregnancy quality and life quality. Secondly, it is the only option for pre-pubertal carriers since OTC can be associated with in vitro maturation of immature oocytes. OTC was primarily used for young carriers planning gonadotoxic therapy for malignant or benign disease [65]. Thirdly, OTC has less effect on the time cycle and therapeutic effect of cancer treatment compared with oocyte preservation. Ovarian tissue harvesting can be performed without delaying oncological therapy and it is even feasible after chemotherapy has begun. OTC can avoid effects caused hormone stimulation of oocyte retrieval since estrogen can lead to malignancy of breast cancer [76, 78]. Despite the discovery of female germ-line stem cells (FGSCs) in the ovary, experiments of FGSCs haven’t been done on human in vitro and the debate on using FGSCs to treat ovarian reproductive is not over yet. Several types of SCs have been used to improve ovarian tissue transplantation by improving graft oxygenation and follicle survival [79, 80]. OTC has a low usage [64] and is currently considered experimental, but pioneers consider it promising [81, 82]. Future research may pave the way for modalities and OTC may become a standard of care for women facing the prospect of sterility from ovarian damage.

#### Preimplantation genetic diagnosis (PGD)

For those who do not wish to pass BRCA gene to the next generation, preimplantation genetic diagnosis method of embryos is recommended. PGD requires couples to undergo IVF procedures, embryos are cultured in vitro tested using PGD for the BRCA mutation before transferring to the female partner. In this manner, only embryos without BRCA mutation are transferred and a mom-to-be does not have to worry her daughter will have to face dilemmas attribute to BRCA mutation [83, 84].

**Fig. 3:**
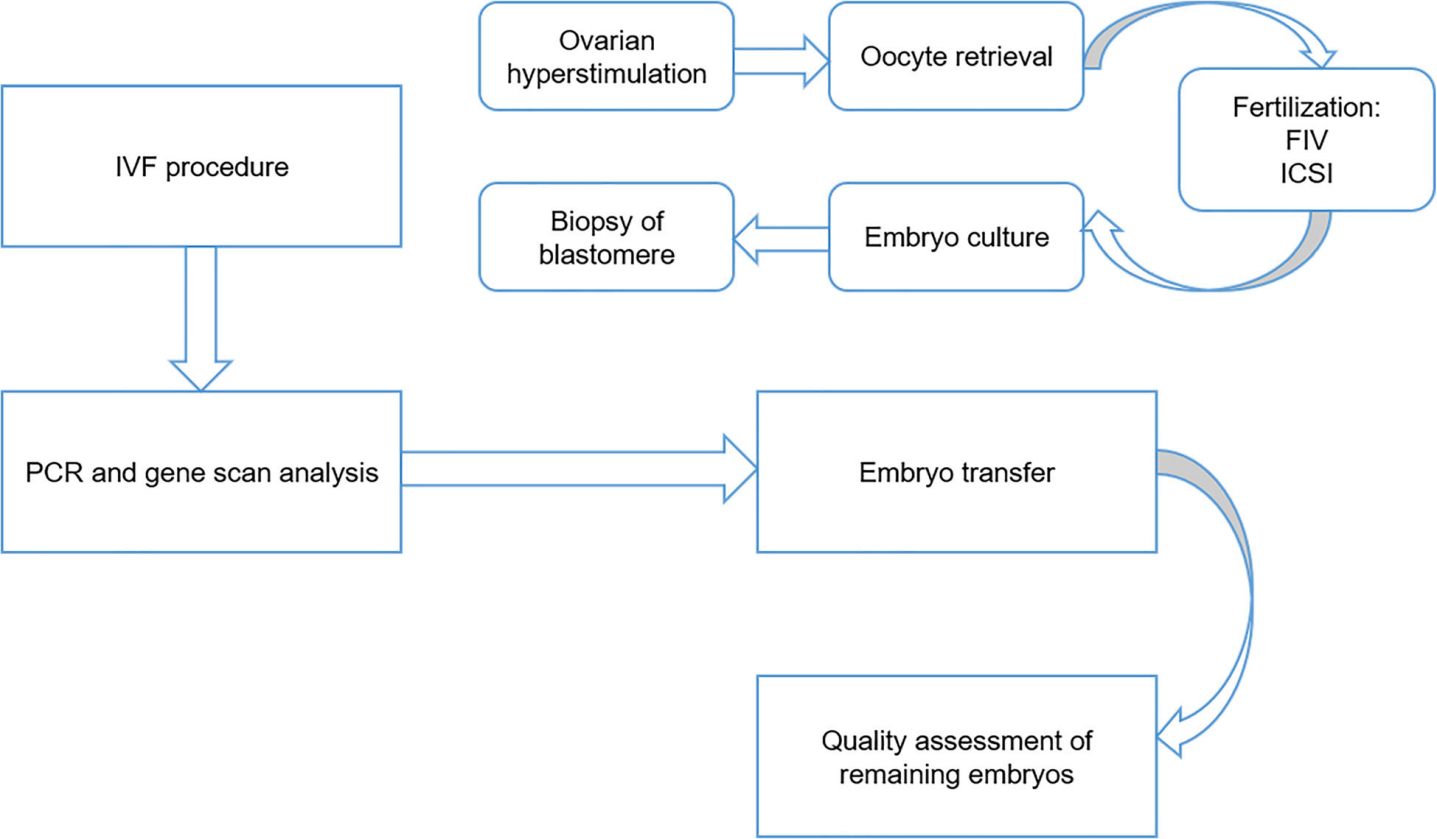
The procedure of PGD

PGD is done by the following process (Fig. 3). COH was performed as described earlier. Oocytes were retrieved under ultrasound guidance. After in vitro maturation, oocytes developed into secondary oocytes with 1 polar body can be fertilized by Fertilization in vitro (FIV) or Intracytoplasmic Sperm Injection (ICSI). FIV refers to keeping the sperm and the oocyte with the cumulus cells in the same medium for fertilization while injecting the sperm into the cytoplasm of the oocyte directly is required in ICSI. Approximately 24 h is needed for final fertilization when secondary oocyte processed meiosis II and 2 polar bodies with 2 nucleuses can be seen microscopically. During incubation, embryo morphology grade was used as one of the parameters to evaluate the embryo quality every day. On the morning of 3 days after fertilization, blastomeres were biopsied from cleavage stage embryos for genetic analysis. From each cleavage-stage embryo, one (4–7 cells) or two blastomeres (> 8 cell stage) were biopsied according to the ESHRE PGD guidelines. Of all cells from cleavage-stage embryos, they can only be biopsied once, as this is a general policy in the university’s IVF center. Usually, two cells are used for ultimate examination in order to increase the number of conclusive genetic results, in case of experiment failure. Afterwards, PCR and gene scan analysis is performed before embryo transfer. Genetic diagnosis of embryos allows researchers to identify the embryos without BRCA mutation and one or two healthy embryo(s) can be transferred into the uterus later. The remaining unaffected, good quality, embryos were cryopreserved for quality assessment afterwards. However, this can only be done when the couple involved consented [85, 86].

PGD is considered a well-established clinical service in many countries for human genetic diseases from early nineties. Numerous single gene disorders have been diagnosed with PGD, and BRCA mutations were successfully tested since 2009. PGD is considered acceptable for BRCA mutation carriers, especially for those who require IVF due to fertility problems. The robust outcomes of the multiplex BRCA tests are in accordance with the ESHRE PGD consortium guidelines in 2010 edition. And according to a clinical research collecting data between 2009 and 2011, 87.2% of embryo transfer has been feasible after PGD [85, 87]. In summary, PGD tests for BRCA mutation carriers are robust in test results. PGD can be easy and quick to implement for a wide range of families hoping to avoid transmission of BRCA1/2 mutation to future offspring.

#### Egg or embryo donation

**Fig. 4:**
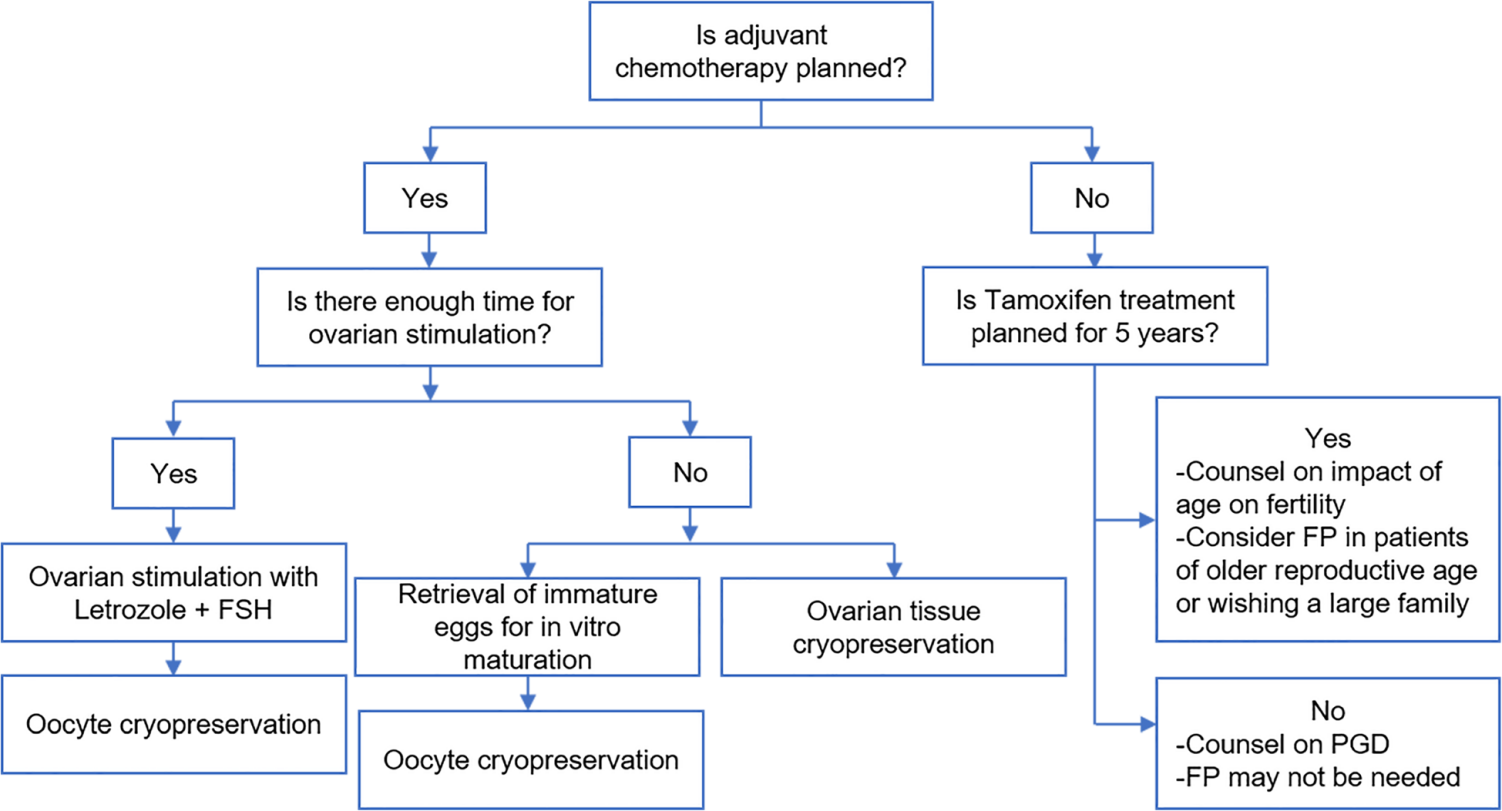
Individualized strategies of FP for BRCA mutation carriers

Egg donation and embryo donation is available even for those who have already undergone prophylactic ovary removal or suffered early menopause because of chemotherapy. This is a backup option of IVF with donor oocytes as a reasonable alternative to freezing their own oocytes (Fig. 4). Egg donation is a preferable option to traditional adoption, but may involve ethic problems in different culture [58].

### Special considerations of special considerations of fertility preservation for woman with BRCA mutation

#### Timeline of fertility preservation

FP should be done before any treatments that may affect fertility as early as possible [88]. To begin with, early referral for FP allows women with BRCA mutation a wider choice since OTC requires a long cycle as early discussed. In a recent study, researchers concluded that FP referral before procedures and surgeries affecting their reproductive potential enables earlier initiation of cryopreservation cycles and multiple FP cycles. Despite the fact that quantity doesn’t guarantee quality, undergoing multiple cycles still have advantages in FP since a larger number of oocytes or embryos were cryopreserved [89]. Furthermore, earlier FP allows earlier pregnancy. Women lose their ability to conceive at a mean age of 41 years old. Finally, oocyte cryopreservation and other FP procedures should be considered prior to preventive surgeries. The risk of carriers develop ovarian cancer increase to 10–21% by the age of 50 years [90]. Recommendations by ACOG 2009 is that women with BRCA1/ 2 mutations should be offered risk-reducing salpingo-oophorectomy (RRSO) by the age of 40 years or when childbearing is complete [91]. Therefore, we suggest early conduction of FP [92, 93].

#### Safety of fertility preservation and pregnancy

Whether FP is safe for woman with BRCA mutation depends on the specific FP procedure and the condition of the carrier. What BRCA mutation carriers care about most might be whether FP would increase their chances of developing breast cancer. According to a case–control study in 1380 matched pairs of women with BRCA1 and BRCA2 mutations, there is no adverse effect of fertility treatment on the risk for developing breast cancer, compared with controls [93, 94]. Additionally, no fetal anomalies or malformations in children were reported in a study of FP in women with breast cancer using embryo freezing with letrozole after a mean follow-up of 40.4 ± 26.4 month [95].

Pregnancy may increase the risk of breast cancer development for BRCA mutation carriers, especially for BRCA2 mutation carriers. A retrospective study showed that, nulliparous carriers were significantly associated with not developing breast cancer when comparing to parous BRCA mutation carriers. Moreover, the number of parous events was significantly associated with the risk of advanced breast cancer stage (stage II or III vs. stage I). Observation showed that the prevalence of the advanced stage was higher in parous women than in nulliparous women and the correlation is more significant for BRCA2 mutation carriers [96]. However, pregnancy is considered safe for breast cancer survivors. According to evidence in the past decade, pregnancy in breast cancer survivors can be considered safe in terms of risks for breast cancer recurrence or death [97]. A systemic study showed for woman at low risk for breast cancer recurrence, pregnancy and breast-feeding after breast cancer was safe and feasible, supported by available data [98]. Moreover, some studies showed a benefit of pregnancy on survival outcomes. These studies support that for women rehabilitated from breast cancer, those who become pregnant are likely to be healthier and less likely to develop a recurrence than women who do not get pregnant [99, 100].

Breast-feeding is adoptable for asymptomatic BRCA mutation carriers. The protective effect of breast-feeding against breast cancer has been shown by studies for BRCA1 mutation carriers. Additionally, no association of breast cancer and breast-feeding was found in BRCA2 mutation carriers [101]. Recent guidelines have suggested avoiding breast-feeding while receiving chemotherapeutic, endocrine or targeted treatment [102]. The interval time of last chemotherapy administration and breastfeeding should be at least three weeks [103]. Breast cancer patients after radiotherapy have decreased milk production along with biochemical changes of the milk [104, 105].

#### Preimplantation genetic test

Performing PGD can avoid the risk of transmission of BRCA mutation, this has opened new perspectives for those with BRCA mutation. It is now considered that PGD tests for BRCA mutation carriers are robust, easy and quick to implement for a wide range of families. The principal of universal protocols is characterized by using highly informative microsatellite markers that are based on genetic linkage. By applying this methodology, there is no need to incorporate the specific familial mutation. The universal protocols make the tests applicable in 90% of couples coming from BRCA mutation carriers [106]. Moreover, the safety of hormonal stimulation and the time frames for PGD planning are essential items that BRCA mutation carriers should take into consideration [107].

#### Psychosocial evaluation before fertility preservation and pregnancy

Psychosocial evaluation before FP and pregnancy is necessary since BRCA mutation carries might face emotional stress of cancer burden, pregnancy and PGD [108]. In a large Gynecologic Oncology Group (GOG) trial, half of the participants (2287 in total), estimated their lifetime ovarian cancer risk greater than 50%, which exceeded the actual risks [107, 109]. In a cross-sectional survey of self-reported BRCA carriers about how knowledge of BRCA status influences their reproductive decisions, 41% of 284 nulliparous women reported carrier status impact their pregnancy choice. The survey also discovered that the majority of respondents (58.7%) thought women with BRCA mutation should be offered with PGD as a choice. However, only 34.8% of respondents would consider undergoing PGD themselves to reduce the risk of transmitting the mutation to their offspring [110]. Moreover, PGD involves discarding normal embryos and this may not be acceptable from an emotional, religious, or ethical point of view [111]. Opinions of health care professionals in Europe and in U.S on PGD for cancer predisposition still varies [83].

#### The choice of hormone for ovarian stimulation

**Fig. 5:**
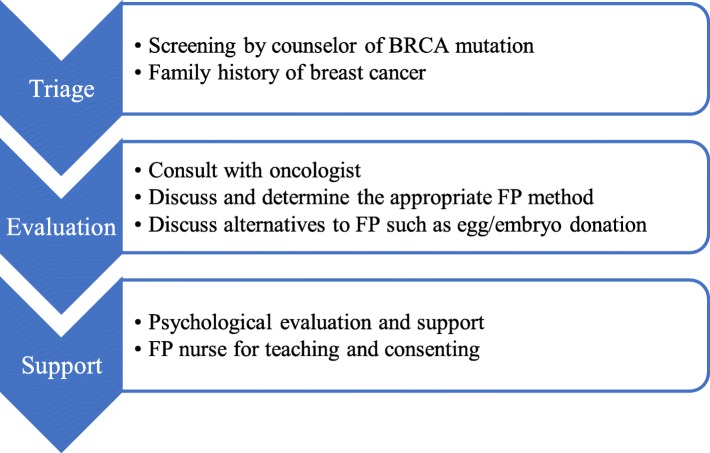
Suggestions for fertility preservation (FP) candidates

For BRCA mutation carriers, a significant concern in preforming FP is the exposure of estrogen during ovarian stimulation. Ovarian stimulation regimens and/or pregnancy increase estrogen levels, this may rise the risk of cancer occurrence/recurrence subsequently (Fig. 5) [107].

According to the ASCO Clinical Practice Guideline update of Fertility Preservation in Patients with Cancer in 2018, aromatase inhibitor–based stimulation protocols are now well established and may ameliorate the concern of FP’s influence on cancer. Current studies do not indicate aromatase inhibitor, including supplemented ovarian stimulation and subsequent pregnancy, resulting increased cancer recurrence [112]. In previous clinical trials, ovarian stimulation with letrozole resulted in larger number of oocytes and embryos as well as lower estrogen levels compared with tamoxifen. It also found that letrozole causes ovarian stimulation along with suppression of estrogen levels at or close to natural cycle levels during a cryopreservation cycle [113]. Recent studies show that using letrozole added to gonadotrophins for COS is safe applying to hormone-sensitive cancer patients as it avoids associated high estradiol levels. This can be another solution of ovarian stimulation provided for BRCA mutation carriers as well [114].

## Conclusions

This paper introduces the impacts of BRCA mutation on carrier’s fertility and the applicability of various available FP solutions. The feasibility of viable FP procedures which includes oocyte cryopreservation, ovarian tissue cryopreservation, PGD, egg or embryo donation depends on particular circumstances carriers face. BRCA mutation carriers should pay special attention to the timeline, safety, use of PGD, psychological factors as well as hormone for stimulation during FP process. Additionally, more research on BRCA germline mutations in different ethnic populations should be done for better BRCA mutation screening and more clinical trials on OTC should be supported as currently data is still far and all between.

## Data Availability

Data sharing is not applicable to this article as no datasets were generated or analysed during the current study.

## Abbreviations

AMH: anti-Müllerian hormone
ATM: ataxia-telengiectasia mutated
B-RST™: Breast Cancer Genetics Referral Screening Tool
COH: controlled ovarian hyperstimulation
COS: controlled ovarian stimulation
dHPLC: denaturing high-performance liquid chromatography
DSB: double strand break
FGSCs: female germ-line stem cells
FIV: Fertilization in vitro
FP: fertility preservation
FSH: follicle stimulating hormone
GOG: Gynecologic Oncology Group
HBOC: hereditary breast/ovarian cancer
HRM: high-resolution melting
ICSI: Intracytoplasmic Sperm Injection
IVF: in vitro fertilization
NGS: next-generation sequencing
OTC: ovarian tissue cryopreservation
PGD: Preimplantation Genetic Diagnosis
POF: premature ovarian failure
RRSO: risk-reducing salpingo-oophorectomy
